# Vomiting of Pregnancy

**Published:** 1880-02

**Authors:** Finley Ellingwood

**Affiliations:** Manteno, Ill.


### Selections.

### VOMITING OF PREGNANCY.

BY FINLEY ELLINGWOOD, M.D., MANTENO, ILL.

In much the larger majority of the cases of vomiting of pregnancy which come under the observation of the general practitioner, various agents are recommended as useful, and the results of their use are in many cases satisfactory. Among these may be mentioned the oxalate and the nitrate of cerium, hydrocyanic acid, pepsine in various combinations, calumba, bismuth, wines of ipecac, and others. But occasionally a physician will meet with a case of this character where all agents are nugatory, the difficulty refusing to yield, however much care may be exercised in the prescribing or administration of them. Such a case came under my observation, and I report it here as containing some points of interest in the treatment of this particularly obstinate and sometimes fatal sympathetic malady.

During the early part of the month of April of last year, I was consulted by Mrs. S., aged about thirty-three years, then but two months pregnant with her fourth child. She informed me that in each of her previous pregnancies the vomiting had been so great and had kept her so reduced that she was confined to her bed the most of the time during the last six months of each term, and that the suffering had been so extreme, notwithstanding she had received the best of treatment, that rather than endure the same for the coming months of the pregnancy, she expressed herself as anxious that I should relieve her of the fœtus. In this her husband heartily concurred.

I told them that if I found the difficulty would not yield after a reasonable treatment, and at any time actually compromised her life, that then I would take the matter into consideration with a consulting physician, but not before. She even at this time was much reduced by excessive vomiting and inability to retain nourishment of any character.

I began a most active treatment and kept it up for about six weeks, thoroughly testing many of the agents usually prescribed, without the least permanent effect. The difficulty assumed an alarming character in its influence upon my patient's health, and I became convinced that something must be done immediately.

At this juncture I remembered having read in Braithwaite's "Retrospect" of 1875 a report by Dr. Copeman, of the British Medical Association, of three cases of this malady which he had relieved completely by carefully dilating the os uteri. I immediately determined, in the exigency of the case, to adopt Dr. Copeman's plan in the treatment of my patient.

Accordingly, on May 26, I made an examination, found the os sufficiently patent to admit the end of my finger. It was considerably puckered and somewhat irritable, but easily dilated. I dilated it as much as I could without using too much force, smoothing out all the wrinkles, and leaving the os tincæ and cervix smooth.

I was somewhat alarmed, upon removing my finger, to find it covered with blood, fearing that I had carried the dilatation too far. I enjoined quiet and a recombent position, and left her. For the next two days, there being no perceptible change in the condition of my patient, she took two or three doses of hydrocyanic acid preparation, which she had taken for a couple of weeks previously with no effect, when the condition became suddenly relieved, and she was restored to perfect health, and went on to the end of the term. I delivered her of a healthy, vigorous child, after a confinement of only four hours. She informed me that all her previous confinements had lasted at least twelve hours. Whether or not this last fact is significant I am unable to say, as several cases have come under my observation where a certain degree of irritability continued until the end of the term, ending in a comparatively easy confinement.

I am convinced that in this case the cause of the irritability was removed by the dilatation, and that the evidence only of the irritation was removed by the acid, as it had produced no effect previous to the dilatation. This method of treatment can be adopted with comparative freedom, as I think that in no case the dilatation need be carried to an extent sufficient to cause the patient to abort, although I think it necessary, however, that caution be exercised, and if premonitory symptoms are discovered, rest must be advised in a recumbent position, and perhaps the exhibition of approximate agents, such as the viburnum prun., will ward off the impending attack.

I do not possess any great degree of confidence in the treatment of this disorder directed to the stomach alone, as the difficulty being sympathetic, it is patent to a careful observer that the treatment should be directed to the cause, and not to the effect of the malady.—*Chicago Medical Times.*

